# Crystal growth and electronic properties of LaSbSe

**DOI:** 10.3390/cryst12111663

**Published:** 2022-11-18

**Authors:** Krishna Pandey, Lauren Sayler, Rabindra Basnet, Josh Sakon, Fei Wang, Jin Hu

**Affiliations:** 1 Materials Science and Engineering Program, Institute for Nanoscience and Engineering, University of Arkansas, Fayetteville, Arkansas 72701, USA; 2 Department of Chemistry & Biochemistry, Missouri State University, Springfield, Missouri 65897, USA; 3 Department of Physics, University of Arkansas, Fayetteville, Arkansas 72701, USA; 4 Department of Chemistry & Biochemistry, University of Arkansas, Fayetteville, Arkansas 72701, USA

**Keywords:** Topological semimetals, electronic transport, heat capacity, layered material

## Abstract

The ZrSiS-type materials have gained intensive attentions. The magnetic version of the ZrSiS-type materials, *Ln*SbTe (*Ln* = Lanthanide), offers great opportunities to explore new quantum states owing to the interplay between magnetism and electronic band topology. Here, we report the growth and characterization of the non-magnetic LaSbSe of this material family. We found the metallic transport, low magnetoresistance and non-compensated charge carriers with relatively low carrier density in LaSbSe. The specific heat measurement has revealed distinct Sommerfeld coefficient and Debye temperature in comparison to LaSbTe. Such addition of a new *Ln*SbSe selenide compound could provide the alternative material choices in addition to *Ln*SbTe telluride materials.

## 1. Introduction

The discoveries of topological semimetals (TSMs) have created promising material platforms to study fundamental physics and develop novel device applications [1-8]. These materials host relativistic fermions with linear energy-momentum dispersions that are analogous to Dirac and Weyl fermions in the field of high energy physics [1,9]. Such Dirac and Weyl states are protected by certain symmetries, and display various exotic properties, such as large magnetoresistance [8], high mobility [8], chiral anomaly [10-13], and surface Fermi arcs [14–18]. Unlike Dirac semimetals (DSMs) and Weyl semimetals (WSMs) where the linearly dispersed bands cross at discrete points in the momentum space, the nodal semimetals (NLSMs) exhibit interesting linear band crossings along one-dimensional loops or lines. Among various NLSMs, the ZrSiS-family compounds have gained intensive attention. It is a large materials family represented by a chemical formula *WHM* (*W*= Zr/Hf/lanthanides; *H*=Si/Ge/Sn/Sb, *M* = O, S, Se,Te) [19–32]. Those compounds crystallize in a layered crystal structure, formed by the stacking of sandwich layers in which the two dimensional (2D) square net of *H*-atoms are sandwiched by *W*-*M* layers. Many *WHM* materials possess tetragonal structures with a space group *P*4/*nmm*, while some exhibit orthorhombic distortions. Two types of Dirac states have been discovered in lots of *WHM* compounds, including the gapless Dirac point states protected by non-symmorphic symmetry, and the slightly gapped Dirac nodal-line states generated by the glide mirror symmetry [19,20,22,31,33]. Those electronic states lead to several interesting properties in *WHMs* such as the surface floating bands [34,35], enhanced electronic correlations [31,32,36], and pressure-induced topological phase transitions [37,38].

The *Ln*SbTe materials represents magnetic version of *WHM* materials [27,28,39–50]. In those compounds, the spin degree of freedom is activated when *Ln* is a magnetic rare earth element such as Ce [28,39,40], Gd [27,41,42], Nd [46,47], Sm [44,45], Ho [48,49], and Dy [50]. Various antiferromagnetic (AFM) ground states have been observed in those *Ln*SbTe compounds [27,28,44,46,48,50]. Non-trivial topology has been demonstrated in several *Ln*SbTe compounds [27,28,44,49–51], which is predicted to be tunable by magnetism when metamagnetic transitions occurs [28]. In addition to magnetism, rich quantum phenomena such as Kondo effects [39,44,46,52], charge density waves (CDW) [40,42,43], enhanced electronic correlations [44,46,49], and possible magnetic frustration [44] have been observed in various *Ln*SbTe compounds. Such abundance of exotic quantum phenomena makes *Ln*SbTe a model platform to investigate topological physics.

In topological materials, spin-orbit coupling (SOC) is a known parameter that affects electronic states, which is relatively easily tunable by elementary substitutions. In *Ln*SbTe, replacing Te with other chalcogen elements is one possible route to vary SOC. Thus far, CeSb(Te_1-x_Se_x_) [40,53] has been reported to show a complex magnetic ordering that results in a possible devil’s staircase in magnetization measurements. In addition, features suggesting a Kondo lattice with a small charge carrier density has been observed in CeSbSe [53]. Extending from tellurides to selenides, those *Ln*SbSe compounds provide additional opportunities to investigate the topological materials and topological physics in *WHM* family. This motivated us to study the previously unexplored non-magnetic *WHM* compound LaSbSe in this work. We found that LaSbSe exhibits metallic behavior in electron transport, with small magnetoresistance and non-compensated charge carriers, which is distinct from the telluride compound LaSbTe.

## 2. Materials and Methods

**Figure 1.**
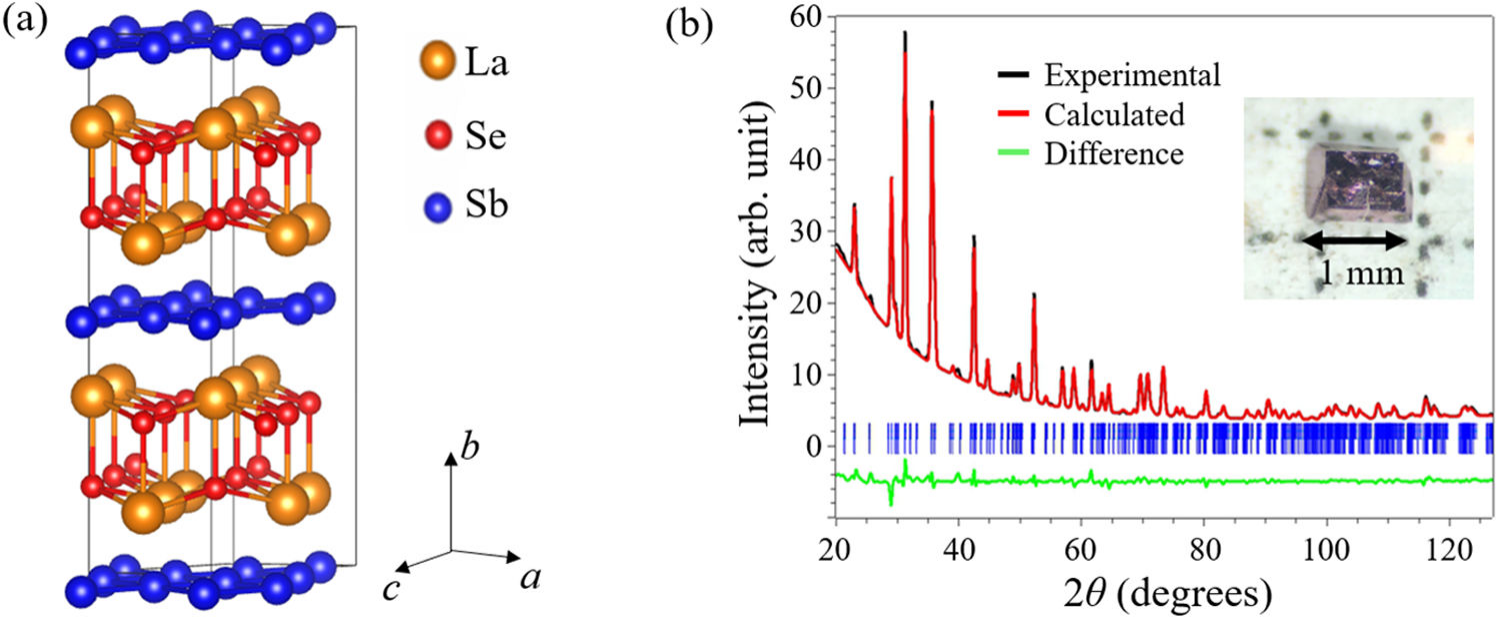
**(a)** Crystal structure of LaSbSe. **(b)** Powder XRD pattern and refinement for LaSbSe. Inset: image of a LaSbSe single crystal.

The LaSbSe single crystals were synthesized by a two-step chemical vapor transport (CVT) method. First, a polycrystalline precursor was prepared by heating the stoichiometric mixture of La, Sb, and Se at 750°C for 2 days. The precursor was then used as the source material for the subsequent CVT growth with selenium tetrachloride as a transport agent. Millimeter-size rectangular crystals with metallic luster were obtained after two weeks’ growth with a temperature gradient from 1000 to 850 °C, as shown in the inset of figure 1(b). The obtained single crystals for LaSbSe are found to be relatively softer and easy to cleave than *Ln*SbTe compounds, which is in line with the lower Debye temperature in comparison to the *Ln*SbTe compounds [39,41,44,46] as will be discussed below. The elemental composition was checked by using energy-dispersive X-ray spectroscopy (EDS). The crystal structure was determined by both powder and single crystal X-ray diffraction (XRD) performed at room temperature by using Rigaku XtaLAB Synergy-S diffractometers. The powder diffraction was performed using Cu Kα radiation, and the single crystal XRD spectrum was collected by using Mo Kα radiation. The refinements of powder and single crystal XRD data were performed by JANA2006 [75]. Electronic transport and heat capacity measurements were performed by using a physical properties measurement system (PPMS).

## 3. Results and Discussions

Stoichiometric *Ln*SbTe compounds crystallize in the tetragonal lattice with nonsymmorphic space group *P*4/*nmm*, in which the Sb square nets are sandwiched between the *Ln*-Te layers [28,43,45,48,50]. This Sb plane can be substituted by Te, resulting in nonstoichiometric compositions *Ln*Sb_1−*x*_Te_1+*x*_ which is accompanied by orthorhombic distortions [41,52]. In addition, vacancies in the Sb layer have also been observed in those nonstoichiometric compounds [43,45]. To resolve the crystal structure of LaSbSe, we performed both powder and single crystal XRD. The crystal structure was solved and refined from single crystal XRD, which was then used as the model structure for a Rietveld refinement with the powder diffraction pattern. The refined lattice atomic parameters are listed in Table 1 and Table 2. Figure 1b shows the powder XRD spectrum and refinement., The refined crystal structure of LaSbSe is a distorted variant of the *P*4/*nmm* structure of *Ln*SbTe. It is pseudo-tetragonal but the Sb square net is distorted, rendering from which a monoclinic structure with a space group of *P*21/*c* can be determined for our LaSbSe. The structure parameters are summarized in Table 1. Compared to a previous report on LaSbSe [76], our refined structure is consistent in space group and atomic positions but evidently different in lattice parameters. A stoichiometric composition, LaSbSe, was also obtained from the structure refinement, which is consistent with the composition analysis using EDS. To further confirm the structure of LaSbSe, we have also resolved the structure using single crystal XRD, from which a consistent crystal structure was obtained as shown in Table 2.

**Figure 2.**
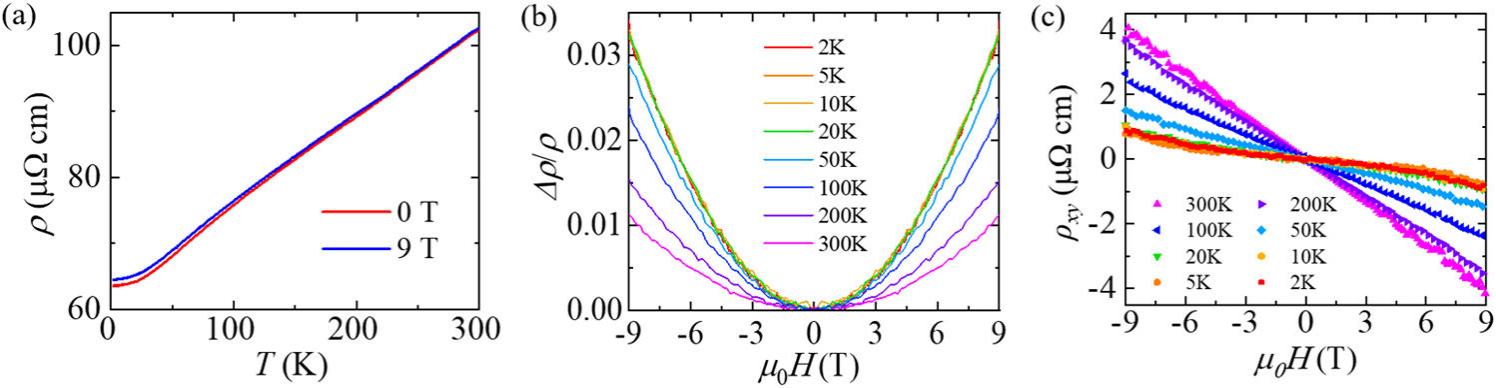
**(a)** Temperature dependent resistivity for LaSbSe, measured with and without applying a magnetic field of 9 T. **(b)** Normalized magnetoresistance at various temperatures for LaSbSe. **(c)** magnetic field dependent Hall resistivity at various temperatures for LaSbSe.

In *Ln*SbTe compounds, both metallic and non-metallic electronic transport behaviors have been observed in temperature dependent resistivity measurements. For example, NdSbTe [46], CeSbTe [39], and SmSbTe [44] exhibit nonmetallic transport (i.e. increase of resistivity upon cooling) with Kondo-like features, whereas the metallic transport (i.e. resistivity decreases with reducing temperature) has been reported in LaSbTe [54], GdSbTe [41,55], HoSbTe [48], and DySbTe [50]. For selenide *Ln*SbSe compounds, metallic transport has also been observed in CeSbSe [53]. As shown in Fig. 2(a), the overall temperature dependence of resistivity for LaSbSe display metallic behavior with decreasing resistivity upon cooling. Despite of the metallic temperature dependence, the residual resistivity ratio *RRR*, i.e., $\rho_{xx}(300\;K)∕\rho_{xx}(2\;K)$, is only 1.6, which is comparable to the non-rare earth *WHM* compound ZrSnTe [56] but significantly lower than ZrSiS [21,57,58], HfSiS [59], and LaSbTe [54], implying bad metallicity for LaSbSe. In addition, resistivity for LaSbSe shows a nearly linear temperature dependence above 80 K, which is independent of the applied magnetic field [Fig. 2(a)]. Similar near-linear resistivity has also been reported in isostructural compound CeSbSe, which is ascribed to anomalous scattering mechanism in bosonic mode [53]. Linear resistivity has also been probed in heavy fermion compounds, Fe-based superconducting metals, cuprates, and twisted bilayer graphene [60–65], which has been attributed to the presence of zero temperature instability.

**Figure 3.**
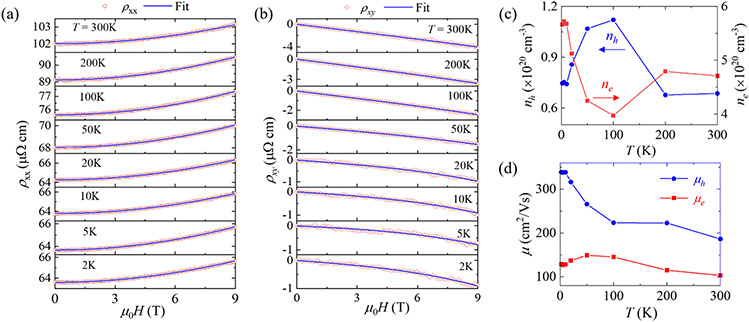
**(a-b)** Simultaneous fits for **(a)** longitudinal resistivity $\rho_{xx}$ and **(b)** transverse resistivity $\rho_{xy}$ using the two-band model. **(c)** Temperature dependent electron and hole carrier densities obtained from the fits. **(d)** Temperature dependent electron and hole carrier mobilities obtained from the fits.

Many TSMs exhibit large positive magnetoresistance (MR) when magnetic field is applied perpendicular to the current direction [66], including the tetragonal non-rare earth *WHM* [21,57–59,67] and the orthorhombic LaSbTe (space group *Pmcn*) [54] which is structurally related to the LaSbSe studied in this work. However, our LaSbSe show very small MR. Figure. 2(b) presents the normalized MR define as [ρ(H)−ρ(0)]∕ρ(0), where ρ(0) and ρ(H) are the resistivity at zero and $\mu_{0}H$ applied field respectively [66]. LaSbSe shows weak positive MR only up to 3.3% at *T* = 2 K and $\mu_{0}H=9\;T$, with a nearly quadratic field dependence. The classical transport theory [68] predicts that in the small field limit, orbital MR due to Lorentz effect exhibits a parabolic field dependence and scales with the square of mobility, i.e., $\text{MR}\propto (\mu H{)}^{2}$ where μ is mobility of charge carriers. The electron-hole compensation prevents MR from saturating, which is also an important factor for large MR. To extract carrier densities and mobilities, we have performed Hall effect experiments. As shown in Fig. 2(c), Hall resistivity $\rho_{xy}$ shows linear field dependence at 300 K, but deviations from linearity can be observed with decreasing temperature. Such non-linearity indicates that both electron- and hole-type carriers contribute to electronic transport in LaSbSe, which has also been observed in non-magnetic ZrSiS-type compounds [21,54,69] but is different from the linear $\rho_{xy}(H)$ seen for a few *Ln*SbTe compounds [39,44,46]. For such a multiband system, carrier densities and mobilities can be obtained by simultaneously fitting both the longitudinal resistivity $\rho_{xx}(H)$ and Hall resistivity $\rho_{xy}(H)$ to a two-band model [66]:

(1) $$\rho_{xx}=\frac{1}{e}\left[\frac{(n_{h}\mu_{h}+n_{e}\mu_{e})+\mu_{h}\mu_{e}(n_{h}\mu_{e}+n_{e}\mu_{h})B^{2}}{(n_{h}\mu_{h}+n_{e}\mu_{e}{)}^{2}+\mu_{h}^{\;\;2}\mu_{e}^{\;\;2}(n_{h}-n_{e}{)}^{2}B^{2}}\right]$$

(2) $$\rho_{xy}=\frac{B}{e}\left[\frac{(n_{h}\mu_{h}^{\;\;2}-n_{e}\mu_{e}^{\;\;2})+\mu_{h}^{\;\;2}\mu_{e}^{\;\;2}(n_{h}-n_{e})B^{2}}{(n_{h}\mu_{h}+n_{e}\mu_{e}{)}^{2}+\mu_{h}^{\;\;2}\mu_{e}^{\;\;2}(n_{h}-n_{e}{)}^{2}B^{2}}\right]$$

where $n_{e(h)}$ and $\mu_{e(h)}$ are carrier density and mobility for electrons (holes) respectively. As shown in Figs. 3(a-b), the two-band model fits $\rho_{xx}(H)$ and $\rho_{xy}(H)$ very well, from which the carrier densities and the mobilities of the electron and hole bands are obtained and shown in Figs. 3(c-d).

As shown in Fig. 3(c), $n_{e}$ and $n_{h}$ are in the order of ~ 10^20^ cm^–3^. Such values are comparable to *WHM*-type topological nodal-line semimetals such as ZrSi*M* (*M* = S, Si, Te). In those materials, carrier densities are lower than conventional metals but much higher than many Dirac nodal-point semimetals, which has been attributed to the nodal-line band structures that possess band crossings along a line near the Fermi level [21,69]. Nevertheless, unlike ZrSi*M* which exhibit nearly perfect electron-hole carrier compensation [21,69], $n_{e}$ and $n_{h}$ in LaSbSe differs a lot, by nearly an order of magnitude at *T* = 2 K (5.66×10^20^ and 7.45×10^19^ cm^−3^ for $n_{e}$ and $n_{h}$ respectively). Similarly, the electron and hole mobilities are also quite different in the entire temperature range from 2 to 300 K. For example, 129 cm^2^/V s for electrons and 339 cm^2^/V s for holes at *T* = 2 K. Such values are significantly lower than LaSbTe [54] and ZrSiS [69], which is in line with the bad metallicity and small magnetoresistance for LaSbSe as stated above. As a comparison, in NdSbTe which show even lower mobility, the metallicity is fully suppressed and the material exhibits non-metallic transport behavior [46].

**Figure 4.**
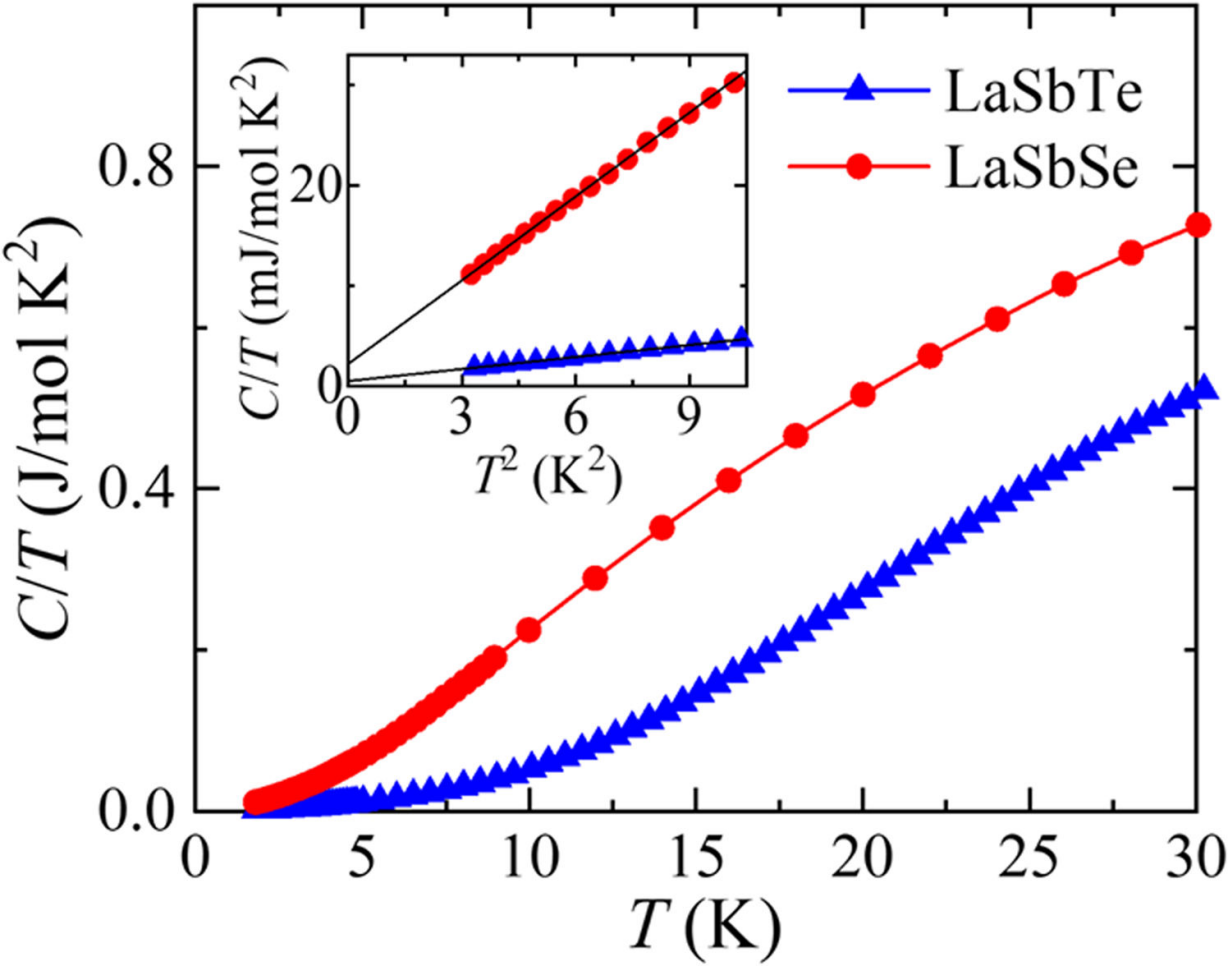
Temperature dependent specific heat divided by temperature *C*/*T* for LaSbSe and LaSbTe. Inset: linear fit for *C*/*T* vs. *T*^2^ at low temperatures for LaSbSe and LaSbTe

Magnetic *Ln*SbTe compounds provide excellent platforms to investigate the new phenomena brought in by magnetic rare earth element *Ln*, such as engineering topological electronic states [28] and electron correlation enhancement [44]. For those telluride materials, non-magnetic LaSbTe provides a good reference to evaluate the effects of magnetism in those magnetic *Ln*SbTe. For example, specific heat measurement is a useful bulk measurement tool to extract information of magnetism and electronic correlations. Magnetic *Ln*SbTe compounds [39,41,44,46,48,50] display clear specific-heat anomalies around the magnetic phase transition temperatures. A few *Ln*SbTe compounds such as NdSbTe [46], SmSbTe [44] and HoSbTe [48] exhibit possible enhanced electronic correlations characterized by large Sommerfeld coefficient *γ*. In previous specific heat studies, LaSbTe has been used as a reference material to precisely evaluate the electronic specific heat for magnetic *Ln*SbTe because of the absence of magnetic specific heat in non-magnetic LaSbTe [44–46]. Similarly, for magnetic selenide materials *Ln*SbSe such as CeSbSe which displays interesting electronic properties such as magnetic Devil’s staircase [40,53] and Kondo lattice behavior [53], the non-magnetic LaSbSe can also act as a good reference for specific heat study. In Fig. 4 we present the temperature dependence of specific heat divided by temperature *C*(*T*)/*T* for LaSbSe. Data for LaSbTe is also provided for comparison. No anomaly is seen in both compounds from 1.8K to 30K, consistent with their non-magnetic nature. Therefore, the total specific heat can be expressed by *C*_tot_ = *C*_el_ + *C*_ph_, where *C*_el_ = *γT* is the electronic specific heat and *C*_ph_ = *βT*^3^ represents the phonon contribution in the low temperature limit. This is clearly reflected by the linear dependence when plotting the low temperature *C*/*T* data against *T*^2^, as shown in the inset of Fig. 4. The linear fits yield Sommerfeld coefficients *γ* of 2.19 and 0.51 mJ mol/K^2^ for LaSbSe and LaSbTe, respectively. Such *γ* values are much lower than magnetic *Ln*SbTe compounds [28,39,44,46], in which large *γ* above 100 mJ mol/K^2^ has been observed and ascribed to the effective mass enhancement due to the presence of flat 4*f* bands near the Fermi level in the magnetically ordered state [44]. The phonon specific heat for LaSbSe and LaSbTe is very different. The fits yield *β* to be 2.78 and 0.39 mJ/ mol K^4^ for LaSbSe and LaSbTe, respectively. This leads to a Debye temperature *θ*_D_ = (12*π*^4^*NR*/5*β*)^1/3^ = 127.93 K for LaSbSe with an atom number per formula *N* = 3 and a gas constant *R* =8.31 J/mol K, which is almost half of 244.53 K for LaSbTe. The suppression of *θ*_D_ for LaSbSe implies weaker interatomic interactions. Indeed, single crystals of LaSbSe are relatively softer than LaSbTe. Modification of *θ*_D_ with chalcogen substitutions Se and Te has been in seen in other materials such as Fe(Se,Te) [70–72] and ZrSi(Se,Te) [73,74], but the reported trends are not consistent. Fe(Se,Te) [73–75] shares a similar doping dependence with LaSb(Se,Te) with enhanced *θ*_D_ up on replacing Se by Te, whereas ZrSi(Se,Te) [73,74] displays an opposite trend.

## 4. Discussion

Authors should discuss the results and how they can be interpreted from the perspective of previous studies and of the working hypotheses. The findings and their implications should be discussed in the broadest context possible. Future research directions may also be highlighted.

## 5. Conclusions

This section is not mandatory but can be added to the manuscript if the discussion is unusually long or complex.

## Figures and Tables

**Table 1.** Room temperature crystal structure determined by powder XRD. Space group: *P*21/*c*. *a* = 4.24437(17) Å, *b* = 18.4271 (4) Å, *c* = 6.0175(4) Å. *β* = 134.971(4)º. *R*_p_ =0.023, *R*_wp_ = 0.032, *R*_exp_ =0.349, *R*(*F*) = 0.060, *χ*^2^ = 0.008.

Atom	Wyckoff	*x*	*y*	*z*	$U_{\mathrm{iso}}$
La	4*e*	‒0.005 (8)	0.35203 (4)	0.242 (4)	0.0105 (3)
Sb	4*e*	0.498 (4)	‒0.00386 (14)	0.750 (3)	0.0039 (3)
Se	4*e*	‒0.006 (11)	0.18612 (7)	0.237 (4)	0.0080 (6)

**Table 2.** Room temperature crystal structure determined by single crystal XRD. Space group: *P*21/*c*. *a* = 4.2512(1) Å, *b* = 18.4342(2) Å, 6.0153(1) Å. *β* = 134.978 (3)º.

Atom	Wyckoff	*x*	*y*	*z*	${U_{\mathrm{iso}}}^{*}∕U_{\mathrm{eq}}$
La	4e	0.49016 (5)	0.352057 (8)	0.24017 (3)	0.00743 (11)
Sb	4e	0.98422 (6)	0.000418 (14)	0.23424 (3)	0.00802 (12)
Se	4e	0.49157 (7)	0.185065 (16)	0.24162 (4)	0.00756 (18)

## Data Availability

The data presented in this study are available on request from the corresponding author.
